# The State’s Greatest Need

**Published:** 1913-08

**Authors:** 


The State's Greatest Need.—"Our National health is physically our greatest National asset," says ex-President Roosevelt in a letter to the Committee of One Hundred of the Association for the Advancement of Science.

It is our greatest asset not only physically, but in every other respect—economically, socially, commercially, morally, mentally and collectively—both National, State and municipal. It is the fundamental and most important interest of a people, a State or a nation, the interest upon which all other interests depend. That nation is not the strongest that has the largest population, for with races as with individuals the strongest and fittest survive, but the one whose units are physically and mentally sound. Yet, strange to say, this greatest and most far-reaching and vital interest is less safeguarded by government than any other, public or private, personal or property.

Ignorance of Conditions.—This paradox is not the result of indifference, but it is because the people and our lawmakers do not know the conditions which are in operation to the detriment of the individual and the deterioration of the race, many of the factors which are preventable and can be removed. Nor do they know the value and scope of sanitary science in preventing and removing those factors. They have not thought and reflected upon the subject, notwithstanding the brilliant example of the prevention of yellow fever by exterminating the fever-producing mosquito, and the conversion of the Panama isthmus country from the most deadly region on earth to one of salubrity, where life is not only possible, but pleasant. And it is almost impossible to educate the people or to get the lawmakers to take any interest in sanitary legislation, however vital. There are so many other things to do that evils which are productive of fearful waste of life and which in the cause of humanity cry aloud for reform are overlooked and neglected, or because the very magnitude of the needed reform discourages all attempts to deal with it. True, efforts are made both by Congress and State Legislatures to deal with certain phases of the subject, but it is inadequate and spasmodic. What is wanted and necessary is a comprehensive law of Congress, supplemented by efficient State laws, covering the entire field of sanitation, and the removal by the strong arm of authority and the strong purse of the general government of the evils which are not only destroying our people by the hundreds of thousands annually, but which are affecting our race integrity in the diminution of the birth rate and the production of feebler and still feebler offspring generation after generation.

Patent Medicines.—One of the greatest evils, one which is far-reaching in its destructive influences, one which flourishes upon the ignorance and credulity of the people. It is the use and abuse of the accursed patent medicines. Many of these contain alcohol, opium, chloral or cocaine. Our daily press, and especially the religious press, fosters and promotes its "interests" by advertisements, many of which are fraudulent on their face and most of which hold out false and delusive hopes by specious promises; many are thinly veiled abortifacients, the use of which should be prohibited by law. The Federal government should deal with the monster evil.

It seems to the writer that the greatest and most imperative duty that the government, State and National, owes to its people is to protect them from these and all other dangers, and especially from the ravages of preventable diseases. To that end National legislation is necessary. It is thought the magnitude and great importance of the problem justifies and demands a National department of public health with a secretary or commissioner in the President's cabinet. In this department there should be separate bureaus covering and dealing with the subdivisions in sanitation; for instance, a bureau of education and schools, of infant hygiene (the greatest mortality is among infants); of general sanitation (under which would come the prevention of water pollution, milk infection, the breeding of flies, local epidemics, etc.); of pure foods and drugs and beverages; of registration (physicians, midwives, drugs and druggists); of quarantine, of immigration, of health information, and, fundamentally, a bureau of vital statistics.

Legislation for Sanitation.—Legislation in the interest of public health is legislation in the interest of public morals and of social economics; for what affects the health of a people adversely affects also the morals and political economies, producing crime, insanity, pauperism and prostitution, and fewer and feebler progeny. It may be that sooner than we expect America will be called upon to measure strength with a virile people who understand and practice sanitary science and who have recently applied its principles in war with such success as to practically nullify the great numerical superiority of their antagonist. That the general government should at once take vigorous control of the situation, and by the enactment and rigid enforcement of a sanitary code, backed by an adequate appropriation, is demanded by every consideration of propriety, public safety, self-defense and national existence. Inordinate wealth, luxury, debauchery, idleness and neglect of the right principles of living render a race unfit, and in the history of the world these have preceded the downfall of the greatest civilizations.

